# Round the Hospitals

**Published:** 1918-02-02

**Authors:** 


### ROUND THE HOSPITALS.

At the Aldwych Club on Tuesday, January 29, Lord Derby, Secretary for War, dealt with Mr. Smallwood's complaint of his treatment in a hospital in France, where his son was a patient (see The Hospital, January 26, pp. 358-9). Lord Derby said: "He had made inquiries into the case of Lieutenant Smallwood. The lieutenant was in hospital suffering from tetanus supervening upon a wound. It was hoped that his life might be saved. On the night of May 21 absolute quiet was essential if he were to recover, and for this reason it was considered advisable by the medical officer that the

☛ Previous articles appeared December 8, 15, 22, and January 5, 12, and 26.

**ROUND THE HOSPITALS—(*continued*).**

parents should not remain. They were asked to withdraw, and were accommodated at a hostel within a minute's walk of the hospital in order that they might be called at any moment. At 5 P.M. on May 22, when the patient was critically ill, an interspinal injection of anti-tetanus serum was given as a forlorn hope, after consultation with the parents and at their wish. The parents were asked to leave the ward for a short time while the operation was being performed, but were brought back when it was over, and remained with their son until he died, at 8.45. There was no question of a regulation turning everybody out at 5 o'clock. That regulation existed, but was never enforced where men were likely to die during the night. The withdrawal of Mr. and Mrs. Smallwood from the ward was entirely in the interests of Lieutenant Smallwood, in order to give him the best possible chance of recovery."

---

The following poem, by the late Lieut.-Col. John McCrae, C.A.M.C., Consultant Physician to the British Armies in the Field, and formerly second in command of No. 3 Canadian General Hospital (McGill), who died on January 28 last at a general hospital abroad, entitled "In Flanders' Fields," appeared in the last issue of the *Canadian Nurse*, which states it is considered by some to be the finest poem of the war. Lieut.-Col. McCrae was a Canadian. He was born at Guelph, Ont., and graduated in arts and medicine at Toronto University. At the outbreak of the war he was Associate-Professor of Pathology at McGill University. His brother is Professor of Medicine at the Jefferson Medical School, Philadelphia. When war was declared, Lieut.-Col. McCrae, who was a veteran of the South African War, was in England, and at once volunteered for the Front. He was attached to an artillery unit of the Service, and saw all the heavy fighting. Later he was put in charge of a base hospital. The poem was originally published in *Punch*:—

"In Flanders' fields the poppies grow
Between the crosses, row on row,
That mark our place; while in the sky
The larks, still bravely singing, fly
Unheard amid the guns below.

"We are the Dead! Short days ago
We lived, felt dawn, saw sunset's glow;
Loved, and were loved; and now we lie
In Flanders' fields.

"Take up our quarrel with the foe!
To you from failing hands we throw
The torch—be yours to hold it high!
If ye break faith with us who die,
We shall not sleep, though poppies grow
In Flanders' fields!"

---

The Ladies' Guild of St. Thomas's Hospital during 1917 has had the misfortune to lose its President, the late Duchess of Connaught, who had held office since 1908, and whose death caused widespread sorrow. H.R.H. the Duke of Connaught has been President of St. Thomas's Hospital for many years, and the acceptance of the office of President of the Ladies' Guild by H.R.H. Princess Patricia will give it new strength and attractive force, calculated to add considerably to the number of voluntary workers rendering personal service to the Guild. Each succeeding year of the war has brought with it greater difficulties for the League than the last. The members have been fired to put out increasing energy continuously, and have so won an ever-growing and generous support for the work from the governors of the hospital. A cheque for £9 13s. 10d. would be welcomed by the honorary secretary, Mrs. A. M. Battle, 49 Harley Street, W. 1, if some generous reader will, as we hope, send it to her to enable the deficiency on last year's accounts to be wiped out. Some idea of the difficulties of keeping the work up to its high standard and increasing its volume each year will be realised when we state that the cost of many materials has risen seriously in price, flannel being now 110 per cent. more than it was in October 1916. Two thousand nine hundred and ten garments were sent in during 1917, and the members are at present at work on 300 flannels and 300 binders for the maternity ward.

---

The immediate wants of the moment are stockings for women and children, and socks for men and boys. All sizes of these are required, with mufflers for men, women, and children, and gifts of any or all these various articles will be very much appreciated and welcomed. The distress of some of the poorer patients leaving the hospital is sometimes terrible, in its forcible contrast with the comfort of others of the same social class who have not been debarred by months of illness from earning the higher wages obtainable during the war. All gifts should be sent to Miss Clerk, Honorary Work Secretary, 25 Sloane Gardens, S.W. The Guild has branches at Great Parndon, Essex, which sent in 219 garments; Denmark Hill, 92 garments; Chesham Bois, 34 garments; Madehurst, Arundel, 66 garments; Harpenden, 32 garments; Croydon, 55 garments. The new members of the Executive Committee are Mrs. Marshall Phillips and Mrs. Fairbairn. Warm testimony is paid to the continuous and valuable support of Mr., Mrs., and Miss Wainwright from year to year, whose interest, it is hoped, will be continued in the work, though the late treasurer has now gone to reside at Godalming.

---

The County Hospital, York, has now agreed to a badge for all nurses on the completion of their training who hold its three years' certificate. The badge is of the size of a 2s. piece in gilt and blue enamel, with the White Rose of York, having in its centre a Red Cross on which there are five small lines. Round the rose in gilt letters are the words "York County Hospital." Each nurse on receiving her certificate for three years' training is presented with this badge. Nurses who trained before

**ROUND THE HOSPITALS—(*continued*).**

December 1917 and hold the three years' certificate, by applying to Miss K. S. Stewart, the matron, enclosing 4s. 6d., the cost price of the badge, and giving the dates of their training, can also obtain it.

We are indebted to Mr. J. Arnold Whitchurch, chairman of the board of management, for the following scale of revised salaries of the nursing staff of the County Hospital, Bedford, which came into operation on January 1, 1918:—

| Post. | Commencing at | By annual Increments. | To Maximum |
|---|---|---|---|
| | £ | £ | £ |
| Matron ... ... ... ... | 125 | — | — |
| Assistant Matron ... ... | 50 | 5 | 60 |
| Men's Ward Sister ... ... | 46 | 4 | 54 |
| Women's Ward Sister ... | 46 | 4 | 54 |
| Night Sister ... ... ... | 46 | 4 | 54 |
| Children's Ward Sister ... | 40 | 4 | 48 |
| Theatre Sister ... ... ... | 40 | 4 | 48 |
| Staff Nurses ... ... ... | 30 | 3 | 36 |
| Probationers ... ... ... | 10 | 5 | 20 |

It is encouraging to find there is a general movement throughout the country in the direction of higher salaries and juster rates of payment for the nursing staff in all hospitals which aspire to be well managed and up to date.

In response to a letter from the Director of Fish Supplies, Ministry of Food, the Steyning Board of Guardians has decided to obtain one barrel of herrings on trial. The idea is to find a substitute for meat by the use of pickled and red (smoked) herrings in the diet of the officials and inmates of the institution. Thanks to the initiative of Miss Carter, the superintendent nurse, the Board has raised the salaries of the probationer nurses. The first, second, and third year salaries have been raised from £10 to £12, from £12 to £15, and from £15 to £20 respectively. We hope that the change in diet will prove as popular and successful as the commendable revision of salaries.

The Infirmary, Lisburn Road, Belfast, has sent £12 to the Nation's Fund for Nurses, of which £7 was contributed by the nurses on the staff, the remainder by the medical staff and the Guardians. We believe the example thus set will be followed

(Continued on page 382.)

### SOME HOSPITAL TYPES OF BRITISH NURSING.

### V.—Sister Children's.

**ROUND THE HOSPITALS—*(continued)*.**

by other training-schools throughout the country. Such a course would be welcomed and backed by the people of all classes. Manchester nurses, we hear, propose to organise a sale of work for the Fund. Preparations are in progress for the coming election of the College Council. The nomination papers will be sent out to the members of the College soon, so that nurses should begin to think whom they wish to represent them, and so prepare themselves for their coming responsibility. Only those candidates who have paid their membership fee will be eligible to receive a nomination paper.

To the war are indirectly due many things which should permanently add to the pleasure of life in these islands and the profitable use of leisure in the pursuit of health and enjoyment. The National Herb Growing Association, of 15 and 16 Verulam Street, Gray's Inn Road, has opened an old, but modernised field, of increasing extent, as the operations of the past season demonstrate. All who associate themselves with the Association have an opportunity to spend some time under ideal conditions in the collection of herbs useful for medicinal and other purposes, which, when collected, are carefully sorted and dried by the finders. By picking and drying some of the commonest field and garden plants, among which are weeds which the farmers and others are glad to be freed from, many people can help to recapture the trade in medicinal herbs. They can at the same time add to their income. Among the simpler varieties we may mention as examples that foxglove leaves, dried so as to retain their colour, yield 4d. to 1s. per lb.; red poppy petals, dried in the shade so as to retain their colour, are paid for by the National Herb Growing Association at the address mentioned at 3s. per lb., which enables 3d. a lb. to be paid to children and others for bringing them to the drier; red rose petals, not white or pink, yield 3s. 6d. a lb.; and marigold petals 1s. 6d.

Most people may be able to gather these and other common field and garden plants, but the industry offers a much wider field for the botanist, as will be realised by the fact that the yield to the National Herb Growing Association during the current year exceeded upwards of twenty tons of various herbs, when dried. So great is the demand in the case of herbs like colchicum, foxglove, dandelion, valerian, poppy petals and heads, elderflowers, blessed thistle, and marigold petals that it this year exceeded the supply. There has been a fair supply and an equal demand for borage, belladonna, blackthorn berries, burdock, hogbean, comfrey, coltsfoot, cherry laurel, celandine, ground ivy, garlic, and yarrow. In each of these cases, from the trade's interest in the herbs, the prices realised have in the majority of cases left a fair profit. We understand that the Association is prepared to extend its operations, and would suggest that those of our readers who contemplate trying their hand at herb-gathering next season should write in good time to the Secretary of the Association asking for particulars, so that they may equip themselves with the necessary knowledge to select the herbs, ascertain in what localities they are most frequently met with, and so become possessed of a health-giving, interesting, and profitable method of rambling in the country during the best season of the year for field rambles. Jam, pickles, and apple-storing, as our readers know, have yielded profitable results, to the satisfaction and honour of those who have taken part in them. We have small doubt that if this week's suggestion proves attractive to a number of our readers they will be able to send us reports next autumn which will be as full of interest to Hospital readers as they should prove profitable to those who intelligently take up herb-gathering for profit and health.

At the annual meeting of the Devon and Cornwall Training-School and Home for Nurses and Three Towns Nursing Association, held at Plymouth recently, reference was made to the difficulty of getting nurses, and, because of the attractiveness of the present conditions of women's labour, even pupils to train for nursing work. The report presented by Miss Baxter Bennett (hon. sec.) stated that in consequence of a serious depletion of staff nurses and pupils the number of cases for 1917 and the visits paid showed a decrease. The Three Towns Association has been at work for nearly a dozen years, and its sphere extends far beyond Plymouth, Devonport, and Stoke, for the Devon and the Cornwall Nursing Associations depend upon it as a training-school for village and district nurses. These two county associations now report, however, that it is " almost impossible to obtain pupils for training, the conditions of female labour being so much more attractive than those of normal times."

The problem thus presented is not confined to Cornwall or Devonshire. It is due to the Great War, which is taxing the energies and strength of the nation, and must be, and ought to be, the first consideration of British people everywhere. In large towns and big centres the demand for workers has brought from their retirement an ever-increasing number of patriotic men who have lived useful lives and had given up active work. If there is a shortage of women to train for village and district nurses, surely each village and district has resident in its midst some of the older women who, like their younger sisters who have gone to the war, might bestir themselves and lend a hand in the nursing, after a short training, until the return of their patriotic juniors.

☛ It is our invariable practice to pay for all items of news which we may publish. The minimum payment is 5s. Paragraphs of about twenty lines in length—that is to say, of 150 words or less—are preferred. Contributions should be addressed to The Editor, The Hospital, 28 and 29 Southampton Street, Strand, London, W.C. 2, and, to be in time for the current week's issue, should reach him by the first post on Mondays.

